# The Period (*per*) Gene Plays an Important Role in Regulating Circadian Oscillation and Ovarian Development in the Ridgetail White Prawn, *Exopalaemon carinicauda*

**DOI:** 10.3390/ani16030513

**Published:** 2026-02-06

**Authors:** Caijuan Tian, Yunhan Feng, Jixuan Zhu, Xuanjian Chen, Wanxin Ma, Panpan Niu, Hao Zhong, Huan Gao, Yuquan Li

**Affiliations:** 1 School of Marine Science and Engineering, Qingdao Agricultural University, Qingdao 266237, China; 2 Jiangsu Key Laboratory of Marine Biotechnology/Jiangsu Key Laboratory of Marine Bioresources and Environment, Jiangsu Ocean University, Lianyungang 222005, China; 3 Faculty of Science, University of British Columbia, 6200 University Blvd, Vancouver, BC V6T 1Z4, Canada; 4 Co-Innovation Center of Jiangsu Marine Bio-Industry Technology, Lianyungang 222005, China; 5 The Jiangsu Provincial Platform for Conservation and Utilization of Agricultural Germplasm, Nanjing 210014, China

**Keywords:** *Exopalaemon carinicauda*, biological clock gene, tissue specificity, circadian oscillation, ovarian development

## Abstract

To explore the function of the period gene (*Ec*-*per*) in *Exopalaemon carinicauda*, we cloned the gene of 4611 bp with a 5′UTR of 201 bp, a 3′UTR of 813 bp, and an ORF of 3597 bp encoding 1198 amino acids. The predicted protein includes two PAS and one PERIOD domain. qPCR analysis revealed that *Ec*-*per* was expressed across all tissues tested at different developmental stages and during both embryonic and larval stages. Moreover, *Ec*-*per* oscillated rhythmically under different conditions of light-to-dark (L:D) ratios, including continuous darkness (0 L:24 D), where changes in the photoperiod influenced amplitude and phase shifts. The knockdown of *Ec*-*per* mRNA significantly reduced the expression of the circadian-related genes timeless (*tim*) and cryptochrome 1 (*cry1*) (*p* < 0.05). This suggests that *Ec*-*per* is an endogenous clock gene that may participate in molecular feedback loops and synergistically regulate the circadian rhythms through interacting with *tim* and *cry1*. RNA interference of *Ec*-*per* also markedly downregulated ecdysone receptor mRNA (*p* < 0.05), suggesting a positive role in the ovarian development of *E. carinicauda.* In situ hybridization further demonstrated that *Ec*-*per* is involved in oocyte proliferation and the accumulation of exogenous nutrients. This study provides new insights for promoting ovarian development and artificial breeding in crustaceans through optimized light-cycle management.

## Simple Summary

This study elucidates the characteristics of the circadian clock gene (period) in the ridgetail white prawn, *Exopalaemon carinicauda* (*Ec-per*). *Ec-per* exhibited rhythmic expression under multiple light–dark (L–D) cycles, including constant darkness, confirming its role as an endogenous oscillator. Functional analysis using RNA interference indicated that *Ec-per*, as an important component of the circadian feedback loop, influenced reproductive physiology by synergistically regulating circadian genes (timeless and cryptochrome1) and upregulating ecdysone receptors. In situ hybridization results further suggested *Ec-per* is involved in oogenesis. Together, these findings highlight the important role of *Ec-per* in circadian rhythm regulation and ovarian development, offering new insights into photoperiod-mediated reproductive regulation mechanisms in crustaceans.

## 1. Introduction

Research on the molecular mechanisms of biological clock genes began with the breakthrough discovery of the *Drosophila* in the 1970s. In 1971, Konopka et al. first identified the period (*per*) gene by screening mutant *Drosophila*, demonstrating that its mutation led to the disruption of circadian rhythms and abnormal pupation cycles [1]. The gene was successfully cloned from *Drosophila* in 1984 [2], marking the beginning of the molecular era in circadian rhythm research. As a core component of the molecular oscillator of the biological clock, the *per* is widely expressed in the central nervous system and peripheral tissues of the organism [3,4,5]. It forms complexes with factors such as TIMELESS (TIM) and CRYPTOCHROME (CRY) to regulate key physiological processes, including circadian rhythm, cell cycle progression, reproductive behavior, and even tumorigenesis [6,7,8].

Circadian rhythms arise from the oscillatory expression of endogenous clock genes, which exhibit autonomous and plastic homeostatic regulation [9]. In addition to the core molecular oscillators encoded by genetics, exogenous timing factors (zeitgeber) include photoperiods, temperature fluctuations, feeding rhythms, and electromagnetic fields [1,10]. Among these, photoperiod is considered the most critical time factor: organisms integrate changes in spectral intensity/duration between day and night, synchronize physiological activities with the external environment [11]. In recent years, research on *per* has focused primarily on mammals and arthropods to elucidate the biological functions and regulatory mechanisms of circadian rhythms. In mammals, the suprachiasmatic nucleus (SCN) of the hypothalamus acts as the central pacemaker regulating the circadian system. The SCN, through the autonomic nervous system, directly or indirectly innervates the pineal gland and adrenal gland: it stimulates nocturnal melatonin secretion from the pineal gland and, via the hypothalamic–pituitary–adrenal (HPA) axis, induces a glucocorticoid peak preceding the active phase. These two hormones serve as key rhythmic signals that not only synchronize the peripheral biological clock but may also provide feedback regulation to the SCN [12]. Regarding the potential indirect effect of feeding behavior on ovarian development, studies indicate that circadian clock genes act as core regulators and rhythmic controllers of ovarian development [13,14]. The disruption of circadian genes leads to a 24 h weight loss under normal dietary conditions, thereby affecting the body’s nutrient and energy metabolism. Collectively, these mechanisms supply essential material and signaling substrates, further modulate the reproductive axis, and target genes within the ovary, ultimately influencing the process and quality of ovarian development. For instance, knockdown of *per* in mice (*Mus musculus*) leads to a loss of light-induced physiological rhythms and a shortened cell division cycle [11,15]. PERIOD (PER) forms positive and negative regulatory loops by interacting with CLOCK, brain muscle ARNT-like protein (BMAL), and TIM, finely tuning the oscillatory rhythm of the biological clock [16,17]. It also regulates circadian rhythms through trimer formation with CRY1 and TIM [18,19].

*Exopalaemon carinicauda*, a medium-sized marine economic shrimp endemic to China, has strong environmental adaptability and high reproductive ability with a short breeding cycle [20,21,22]. Females spawn annually from March to October. Nevertheless, the large-scale artificial seedling production for this species has not been successfully established, and the seedlings are still largely obtained from fertilized broodstock captured in natural waters [23,24]. In mammals and insects, studies have shown that circadian genes play a crucial role in reproduction [25,26,27]. Although research on clock genes in crustaceans has progressed in species such as *Macrobrachium nipponense* [28,29], *Litopenaeus vannamei* [6], and *Euphausia superba* [30,31], focusing on reproductive strategies in shrimp remains relatively limited. To explore the role of clock genes in the ovarian development of *Exopalaemon carinicauda*, this study cloned the *per* cDNA sequence and investigated the expression patterns of the *per* gene in *E. carinicauda* under varied light colors and photoperiods. Our results found that the *per* gene participates in regulating the circadian rhythm and positively affects ovarian development. These findings provide new theoretical insights into light-regulated reproductive regulation in crustaceans.

## 2. Materials and Methods

### 2.1. Ethics Statement

All experimental procedures in this study adhered to principles of laboratory animal care and use. Although *E. carinicauda* is an invertebrate of small size and not subject to review by institutional animal ethics committees, we still made efforts to minimize potential pain and stress throughout the experiments.

### 2.2. Experimental Materials

Healthy *E. carinicauda* from the self-breeding families of the research group were identified and selected for study. Prior to the experiments, shrimp (body length of 5.11 ± 0.41 cm, body weight of 2.10 ± 0.36 g) were acclimated for one week in 12 L:12 D in aerated tanks (50 × 40 × 30 cm) with a water temperature of 25 °C, salinity of 26, and pH of 8.0. Shrimp were fed daily at 08:00 and 18:00 with commercial feed equivalent to 3% of their body weight.

Samples of 24 individuals were collected at embryonic and larval stages, including: fertilized eggs, Zoea stages I–III, post-larval stage, adult stage, gonadal maturity stage, and postpartum recovery period. Furthermore, nine tissues from 9 shrimp were also collected, including: eyestalk, gill, heart, hepatopancreas, stomach, intestine, muscle, ovary, and ventral nerve cord at the stages of the adult, gonadal maturity, and postpartum recovery, following the method described in Ref. [32]. Ovarian tissues were further collected according to the five defined developmental stages [33]. All samples were taken with at least three biological replicates for each stage or tissue type (*n* ≥ 3).

### 2.3. Total RNA Extraction and cDNA Synthesis

Total RNA was extracted from all the samples using Trizol reagent (TransGen, Beijing, China) according to the manufacturer’s protocol. cDNA was synthesized using the HiScript III RT SuperMix for qPCR (+gDNA wiper) (Vazyme Biotech Co., Ltd., Nanjing, China), diluted for subsequent experiments, and then stored at −20 °C.

### 2.4. Cloning of Ec-per cDNA

The *Ec-per* cDNA sequence was obtained from the transcriptome database [34] of *E. carinicauda* with 5′/3′-RACE amplification kits (Takara, Beijing, China) along with gene-specific primers (as shown in Table 1). The RACE PCR was conducted following the manufacturer’s instructions of the Ex Taq kit (Takara).

### 2.5. Bioinformatics and Phylogenetic Analyses of Ec-per

The open reading frame (ORF) and encoded amino acid sequences of the PER were identified using ORF Finder (http://www.ncbi.nlm.nih.gov/gorf/gorf.html (accessed on 3 February 2026)). The theoretical isoelectric point and molecular weight were calculated by DNASTAR (V 17.5) software. The amino acid sequences of the PER from different species were multiple aligned. A phylogenetic tree was constructed with MEGA (version 11) software using the neighbor-joining method, with 1000 bootstrap replications [35].

### 2.6. qRT-PCR

The qRT-PCR primers used for the gene expression analysis are listed in Table 1. qPCR amplification was performed on a StepOne Plus system using SYBR Premix Ex Taq II kit (TaKaRa, San Jose, CA, USA) according to the manufacturer’s protocol, with 100 ng of cDNA from each sample as template. Relative expression levels were calculated using the 2^−ΔΔCt^ method [36] and are presented as mean ± standard deviation (SD) from at least three independent experiments. The selection of 18S rRNA as the reference gene was based on its demonstrated stability in prior validations performed by our research team [21,23,24] (*p* > 0.05). All statistical analyses were conducted with GraphPad Prism (version 10). Differences between the two groups were assessed using an independent-samples *t*-test, while comparisons among three or more groups were performed by Analysis of Variance (ANOVA) followed by Duncan’s multiple comparisons. *p* < 0.05 indicates a significant difference.

### 2.7. Experiments on Different Light Colors and Photoperiods

The experimental design comprised two parts: (1) Light-color experiment: A total of 180 shrimp were distributed into white, blue, and red light groups under continuous illumination (15 W, 1500 Lx). (2) Photoperiod experiment: 200 shrimps were randomly assigned to five light:dark ratio environments (0 L:24 D, 8 L:16 D, 12 L:12 D, 16 L:8 D, and 24 L:0 D). In both experiments, the eyestalks were taken at 0 h (the control group), 3 h, 6 h, 9 h, 12 h, 15 h, 18 h, 21 h, and 24 h. Each experiment included three parallel groups, and six replicate samples were taken at different time points.

### 2.8. RNA Interference

The siRNA primers of *per* (Table 1) were designed using the Thermo Fisher (Waltham, MA, USA) primer design online tool (http://rnaidesigner.thermofisher.com/rnaiexpress/design.do (accessed on 3 February 2026)), and were synthesized with TR102-T7 RNAi Transcription Kit (Vazyme, Nanjing, China) following the manufacturer’s instructions. A total of 200 shrimp were randomly assigned to the interference and control groups (three replicate groups/group). The siRNA (4 µg/g) or an equivalent volume of normal saline was injected into the pericardial cavity of shrimp in the respective groups. Eyestalk samples were collected at 4 h, 8 h, 12 h, 16 h, 20 h, and 24 h post-injection; ovaries tissues were also taken at 12 h and 24 h. Each time point had greater than three independent biological replicates.

### 2.9. Overexpression Experiment

According to the *Ec-per* core sequence, homologous recombination primers were designed using the CE Design software (V1.03) (as illustrated in Table 1). The pSPT18 plasmid was digested with *Bam*HI and *Eco*RI, purified, and ligated with PCR-amplified and purified homologous recombination fragments. After successful sequencing (Sangon, Shanghai, China), the *per* mRNA for overexpression was transcribed in vitro using Easy Cap T7 Co-transcription Kit with CAG Trimer (Vazyme, China) according to the instructions. A total of 200 shrimp (each group includes 3 parallel groups) were injected into the pericardial cavity with *per* mRNA (4 µg/g) or an equal volume of normal saline, respectively. At least three duplicate samples were collected from different time points.

### 2.10. In Situ Hybridization (ISH)

According to the *Ec-per* core sequence, homologous recombination primers were designed using the CE Design software (V1.03). Probes primers for generating the SP6/T7 promoter for in situ hybridization (ISH) are listed in Table 1. The pSPT18 plasmid vector was digested with *Bam*HI and *Eco*RI and then purified. After the preparation of homologous recombination primers for PCR amplification and purification, the recombination fragments were ligated into the vector. After successful sequencing (Sangon), the recombination vector was linearized with *SP6/T7* probes primers and purified, and used as a template for mRNA probe synthesis using the Biotin RNA Labeling Kit (Beyotime, Shanghai, China) according to the manufacturer’s protocol. In vitro transcription with SP6 and T7 polymerases produced sense (negative control) and antisense (positive control) probes, respectively. Ovarian tissues from 20 individuals were fixed in 4% PFA for 24 h at 4 °C. The section of Hematoxylin–Eosin staining [37] was used for the control group. ISH was carried out with modifications to a previously described protocol [38]. Deparaffinized sections were incubated with 3% H_2_O_2_ for 10 min to remove endogenous peroxidase. Then it was digested with Proteinase K (20 ug/mL) at 37 °C for 10 min, prehybridized at 38 °C for 2–4 h, and hybridized with the Biotin-labeled (1:500) mRNA probe (1 ug/mL) against *Ec-per* mRNA overnight at 38 °C. Sense probes transcribed from the SP6 promoter served as the negative control, and probes targeting the T7 promoter were used as the positive control. Each group included three independent biological replicates. Finally, probe binding was visualized using DAB substrate (Beyotime, Shanghai, China).

## 3. Results

### 3.1. Cloning and Phylogenetic Analysis of the per cDNA

**Figure 1:**
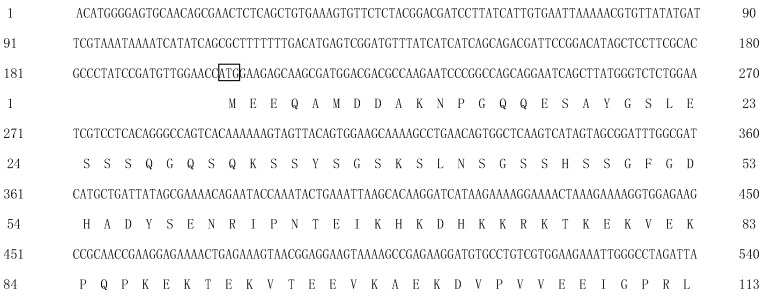

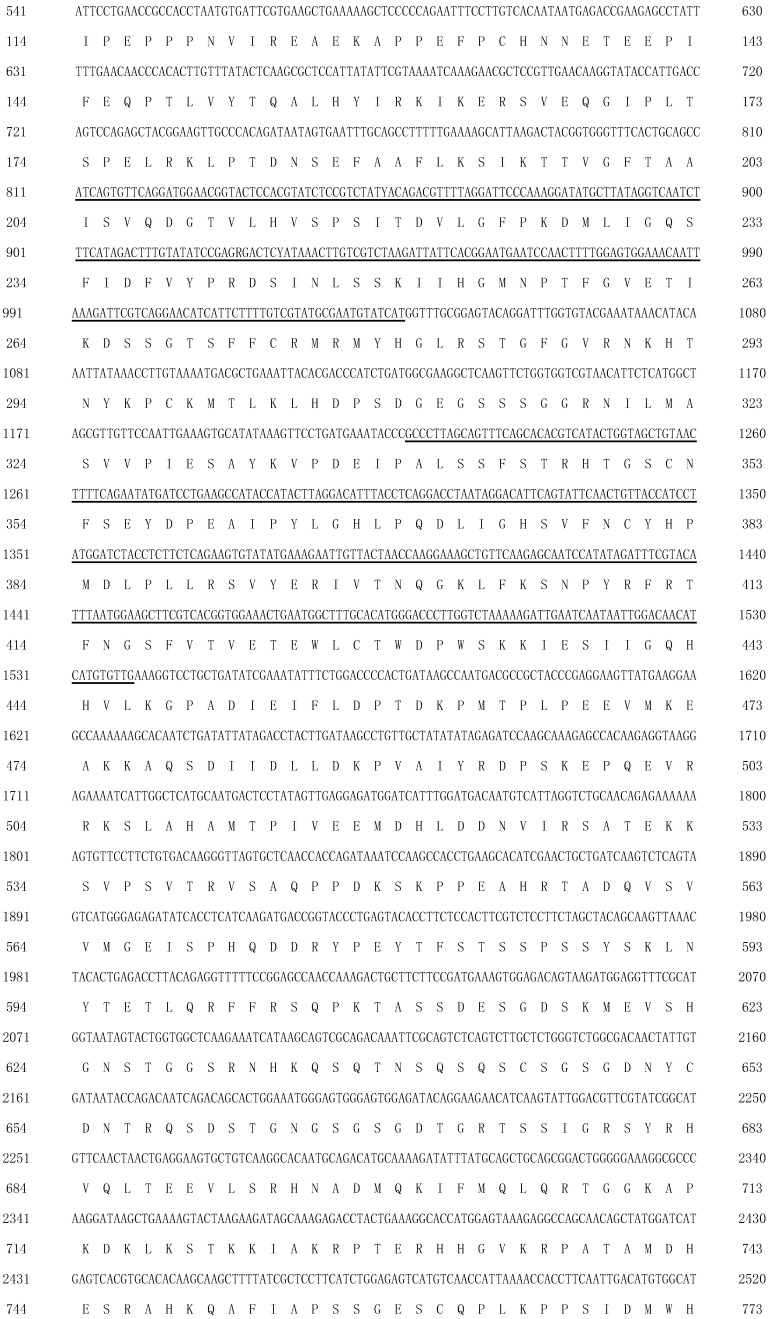

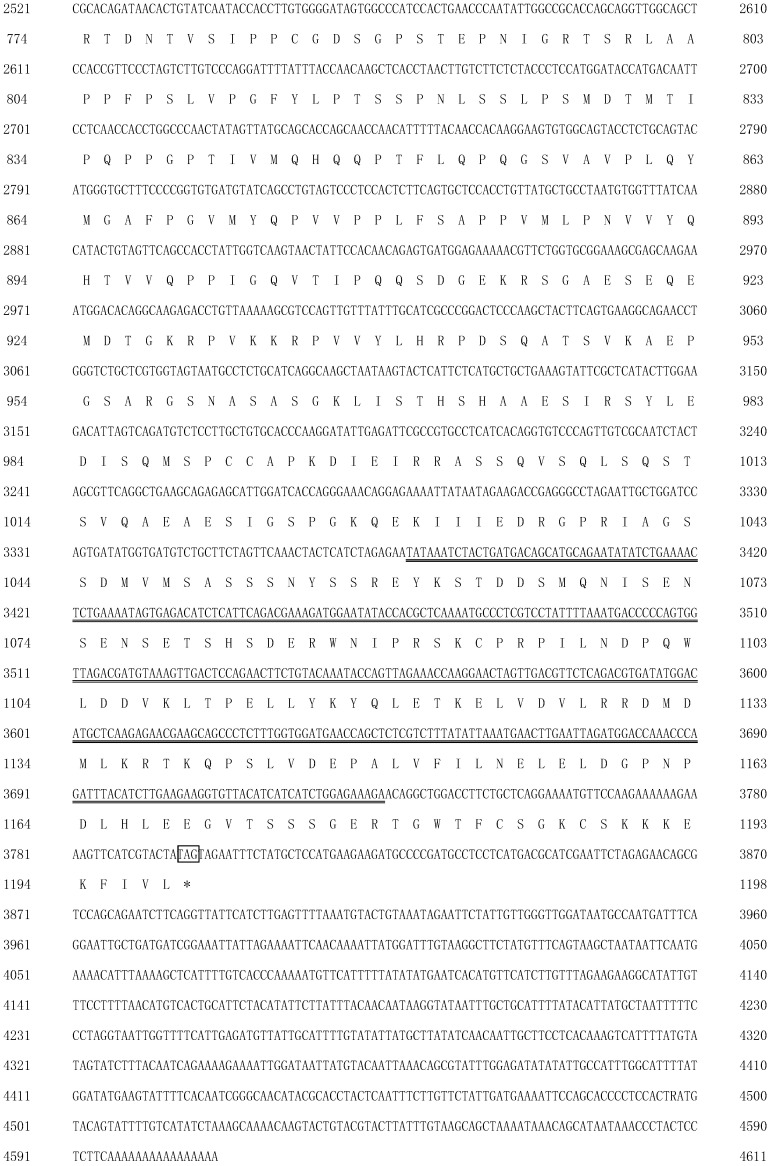
The *per* cDNA sequence and amino acid sequence of *E. carinicauda.* The start codon (ATG) and the stop codon (TAG) are indicated by the box; the single-underlined parts indicate the PAS domain, and the double-underlined regions represent the PER domain.

**Figure 2:**
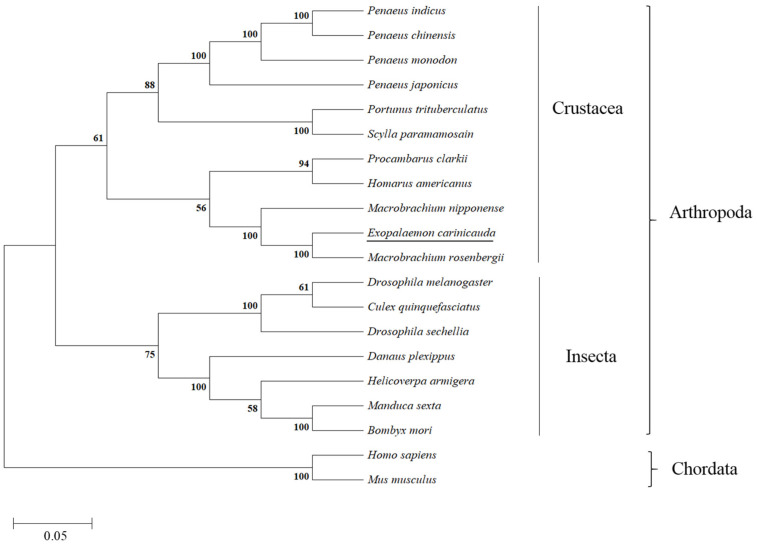
Phylogenetic tree based on PER amino acid sequences of *E. carinicauda* and other species. Notes: *Penaeus indicus* (XP_063601074.1), *Penaeus chinensis* (XP_047479160.1), *Penaeus monodon* (XP_ 037802358.1), *Penaeus japonicas* (XP_042864689.1), *Portunus trituberculatus* (XP_045105926.1), *Scylla paramamosain* (XP_063888150.1), *Procambarus clarkia* (XP_069167826.1), *Homarus americanus* (AWC08578.1), *Macrobrachium nipponense* (XP_064105349.1), *Macrobrachium rosenbergii* (XP_066986239.1), *Drosophila melanogaster* (NP_525056.2), *Culex quinguefasciatus* (XP _038114650.1), *Drosophila sechellia* (XP_032580201.1), *Danaus plexippus* (XP_061382449.1), *Helicoverpa armigera* (XP_049704598.1), *Manduca sexta* (XP_030039609.2), *Bombyx mori* (NP_001036975.1), *Homo sapiens* (NP_002607.2), and *Mus musculus* (XP_006532543.1). *E. carinicauda* is the underlined part. Numbers represent the bootstrap values, calculated by MEGA 11 software. A scale of 0.05 represents evolutionary distance.

The *per* gene sequence has been submitted to the GenBank database (GenBank accession number PX552620). The *Ec-per* cDNA is 4611 bp in length, consisting of a 201 bp 5′-untranslated region and an 813 bp 3′-untranslated region. The ORF of *per* is 3597 bp, encoding a 1198 bp amino acid protein. The predicted molecular mass of 132.6 kDa, with a theoretical isoelectric point of 6.46 (Figure 1). The PER contains the two PAS (Period-Arnt-Single-minded) binding domains at 204~279 AA and 340~446 AA, respectively, and one PER domain at 1060~1178 AA. Phylogenetic analysis further reveals that the *Ec-per* has the closest phylogenetic relationship with *Macrobrachium rosenbergii*, followed by that with *M. nipponense* (Figure 2).

### 3.2. Expression Characteristics of Ec-per at Different Developmental Stages

**Figure 3:**
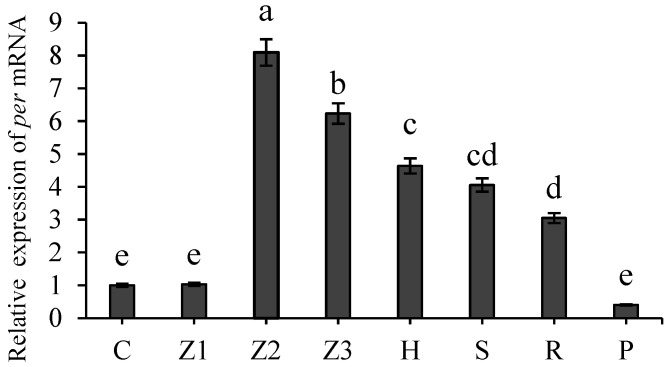
Expression of the *per* gene at different developmental stages of *E. carinicauda*. Notes: C—fertilized eggs, Z1–Z3—Zoea larval stages I–III, H—post-larval stage, S—adult shrimp stage, R—gonadal maturity stage, P—postpartum recovery period. Data are presented as mean ± SD (biological replicates *n* ≥ 3). Statistical analysis was performed using ANOVA followed by Duncan’s test. Different lowercase letters indicate significant differences (*p* < 0.05).

The expression profile of *Ec-per* was further examined during the embryonic and larval stages. qPCR analysis revealed low transcript levels at stages of fertilized eggs and Zoea larvae I, with a significant peak observed at Zoea II (*p* < 0.05) (Figure 3). As development proceeded, the *per* expression declined markedly (*p*  <  0.05), reaching its lowest level in the postpartum recovery period.

### 3.3. Tissue Distribution of Ec-per mRNA

**Figure 4:**
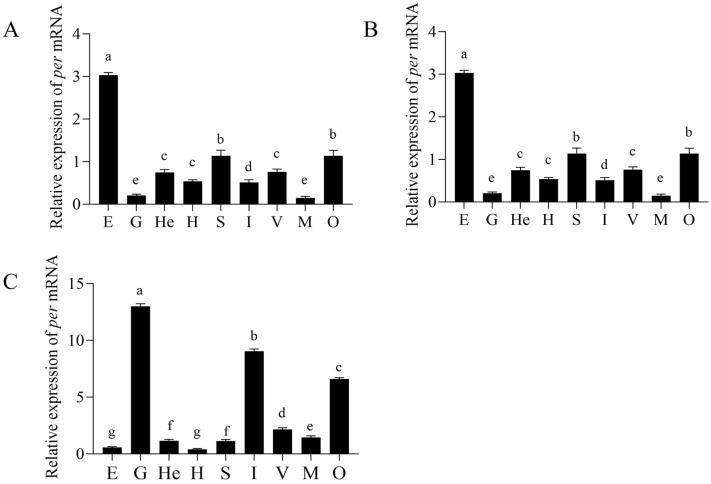
The *per* gene expression in various tissues at different developmental stages. (**A**) Adult shrimp stage, (**B**) gonadal maturity stage, (**C**) postpartum recovery period. E—Eyestalk, G—gill, He—heart, H—hepatopancreas, S—stomach, I—intestines, V—ventral cord nerves, M—muscles, O—ovary. All data are presented as mean ± SD (biological replicates *n* ≥ 3). Statistical analysis was performed using ANOVA followed by Duncan’s test. Different lowercase letters indicate significant differences (*p* < 0.05).

The expression profiles of *Ec-per* across tissues were further analyzed during the different adult developmental stages. *Ec-per* expression varied significantly among tissues at each stage (Figure 4). At the adult shrimp stage, *Ec-per* transcripts were primarily enriched in the heart and intestine (*p* < 0.05), followed by the gill, stomach, and ventral cord nerves. Expression was relatively low in the hepatopancreas and muscle, and the lowest in the eyestalk and ovary. During gonadal maturation, the highest expression level was detected in the eyestalk (*p* < 0.05), while other tissues showed lower expression, with the gill and muscle exhibiting the lowest levels. In the postpartum recovery period, *per* mRNA accumulated most abundantly in the gill, intestine, and ovary, significantly higher than in other tissues (*p* < 0.05), whereas the hepatopancreas showed the lowest expression.

### 3.4. Ec-per Expression in Response to Different Light Colors and Photoperiods

**Figure 5:**
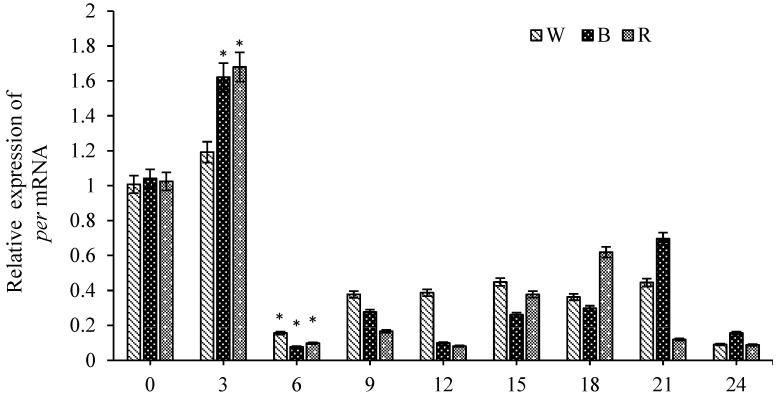
Expression characteristics of *per* in *E. carinicauda* at 0 h, 3 h, 6 h, 9 h, 12 h, 15 h, 18 h, 21 h, and 24 h under different light colors. Notes: W—white; B—blue; R—red. Data are presented as mean ± SD (biological replicates *n* = 6). Statistical analysis was performed using ANOVA followed by Duncan’s test. * at 3 h indicates a significant difference compared to the white light group (0 h) (*p* < 0.05). * at 6 h denotes a significant difference compared with the 3 h group (*p* < 0.05).

The effect of different light colors on the expression of the *per* gene in *E. carinicauda* was studied (Figure 5). Following blue and red light exposures, *per* expression significantly increased, peaking at 3 h (*p* < 0.05), and subsequently exhibited low-amplitude oscillations under all three light conditions.

**Figure 6:**
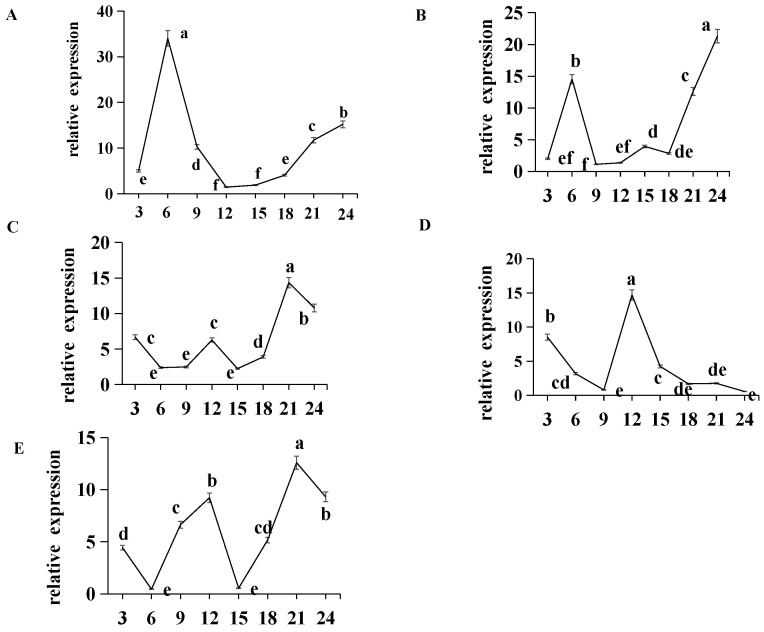
Expression patterns of *per* show rhythmic oscillations in the eyestalk by qPCR analysis. (**A**) 0 L: 24 D; (**B**) 8 L: 16 D; (**C**) 12 L: 12 D (**D**) 16 L: 8 D; (**E**) 24 L: 0 D. Data are presented as mean ± SD (biological replicates *n* = 6). Statistical analysis was performed using ANOVA followed by Duncan’s test. Different lowercase letters indicate significant differences (*p* < 0.05).

We further examined the expression pattern of *per* in the circadian rhythm of *E. carinicauda.* qPCR results indicated that *Ec-per* expression exhibited regular oscillations under various light-to-dark (L:D) ratios, including continuous darkness (0 L:24 D) (Figure 6A). Although the oscillatory rhythm of *per* remained relatively stable under constant darkness, both the amplitude and phase of oscillation shifted under varying photoperiods. Specifically, compared to the complete darkness, the oscillation phase shifted backward by 3 h under an 8 L:16 D photoperiod, and further delayed by 9 h under 16 L:8 D. Under constant light (24 L:0 D), the phase shift extended to 15 h (Figure 6B–E).

### 3.5. RNA Interference and Overexpression of Ec-per and Expression of Related Genes

**Figure 7:**
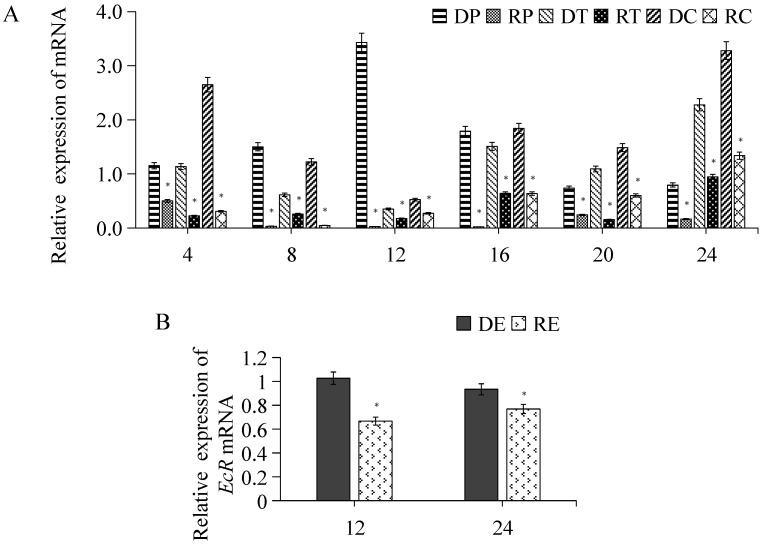
Relative expression of circadian clock genes after *per* mRNA interference. (**A**) Relative expression of *per*, *tim*, and *cry*1 in the eyestalk of control (DP, DT, and DC) and interference group (RP, RT, and RC) at 4 h, 8 h, 12 h, 16 h, 20 h, and 24 h, respectively. (**B**) Relative expression of *EcR* in the ovary of control (DE) and interference (RE) groups at 12 h and 24 h, respectively. Data are presented as mean ± SD (biological replicates *n* ≥ 3). Statistical analysis was performed using ANOVA followed by Duncan’s test (**A**). Student’s *t*-test was also conducted (**B**). * denotes a significant difference compared to the control group (*p* < 0.05).

After interfering with *per* expression, its mRNA level in the eyestalk was significantly reduced (*p* < 0.05) compared to the control group (interference efficiency >60%), confirming effective knockdown. Other core clock genes, including *tim* and *cry1*, also showed significantly decreased expression (Figure 7A). Further analysis of ovarian development revealed that the expression level of the Ecdysone receptor (*EcR*), which is involved in gonadal development, was also significantly lower than that of the control group at 12 h and 24 h post-interference (*p* <0.05) (Figure 7B).

**Figure 8:**
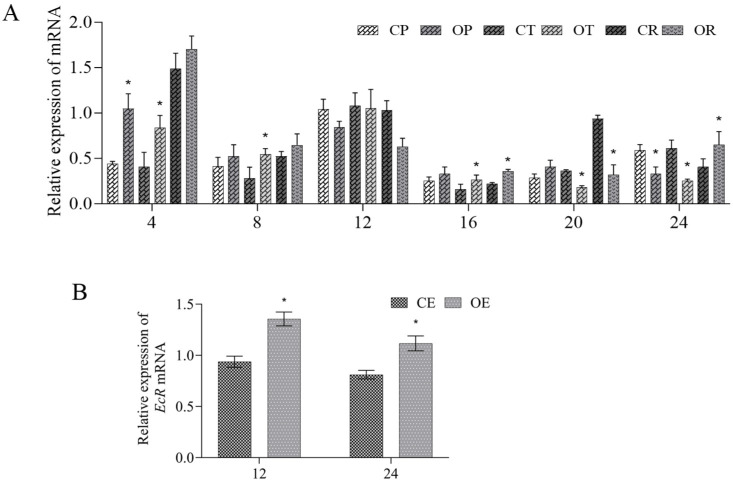
Relative expression of circadian clock genes after the *per* mRNA overexpression. (**A**) Relative expression of *per*, *tim*, and *cry*1 in the eyestalk of control (CP, CT, and CR) and overexpression group (OP, OT, and OR) at 4 h, 8 h, 12 h, 16 h, 20 h, and 24 h. (**B**) Relative expression of *EcR* in the ovary of control (CE) and overexpression (OE) groups at 12 h and 24 h. Data are presented as mean ± SD (biological replicates *n* ≥ 3). Statistical analysis was performed using ANOVA followed by Duncan’s test (**A**). Student’s *t*-test was also conducted (**B**). * denotes a significant difference compared to the control group (*p* < 0.05).

After the overexpression of *per* mRNA, qPCR results indicated that the expression of *per* was significantly lower at 4 h (overexpression efficiency >60%, *p* < 0.05), confirming successful overexpression (Figure 8A). Interestingly, its expression subsequently declined relative to the control at 8 h, 12 h, and 16 h, before increasing again at 20 h and 24 h. Furthermore, the *EcR* mRNA level was significantly higher than that of the control group at 12 h and 24 h post-overexpression (*p* < 0.05) (Figure 8B).

### 3.6. Effect of per on the Ovarian Development of E. carinicauda

**Figure 9:**
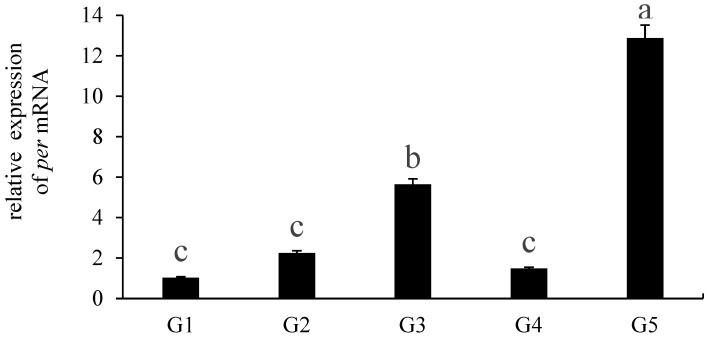
Expression of *Ec-per* at different gonadal developmental stages. G1—proliferation period; G2—small growth period; G3—great growth period; G4—mature period; G5—postpartum recovery period. Data are presented as mean ± SD (biological replicates *n* ≥ 3). Statistical analysis was performed using ANOVA followed by Duncan’s test. Different lowercase letters represent significant differences (*p* < 0.05).

**Figure 10:**
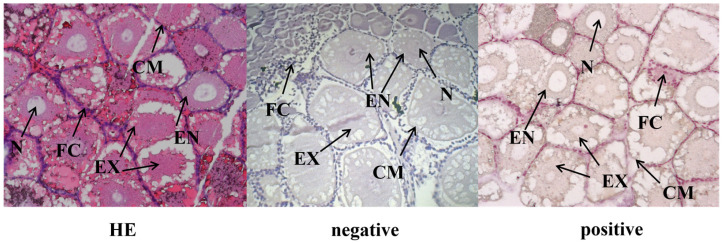
Effects of *per* on the ovarian development of *E. carinicauda*. EN—endogenous vitellogentic oocytes; EX—exogenous yolk synthesis oocytes; N—nucleus; CM—cytoplasmic membrane; FC—follicle cell. Biological replicates *n* = 3. Scale bar: 100 μm.

Expression characteristics of *per* were further examined across five ovarian developmental stages. Our results showed that *per* expression was significantly higher at the G3 and G5 stages than at other stages (*p* < 0.05), while no significant differences were observed at the G1, G2, and G4 stages (Figure 9). ISH was used to localize the *per* mRNA in the ovary of *E. carinicauda* (Figure 10). The strongest signal of *per* was detected in oocytes, including both exogenous yolk synthesis oocytes and endogenous yolk synthesizer oocytes, suggesting a potential role in the nutrient accumulation during oocyte maturation.

## 4. Discussion

### 4.1. The per Gene Exhibits Tissue-Specific and Developmental Stage-Dependent Characteristics

In this study, the *per* gene was first cloned from *E. carinicauda*. Sequence analysis revealed that the encoded PER protein contains two PAS domains and one PER domain. As critical structural motifs for the synergistic interaction of clock proteins [39,40,41], PAS domains are evolutionarily conserved across species [3,42] and have also been identified in zebrafish and mammals [43,44,45].

Expression analysis showed that *Ec-per* transcript levels were significantly higher during the Zoea II larval stage compared to the other developmental stages (*p* < 0.05), consistent with the findings in zebrafish [11], suggesting a conserved role for *per* in the larval development. Tissue-specific expression profiling in adult shrimp indicated high *Ec-per* expression in the heart and intestine, which may support the gonadal development by enhancing physical activity and energy metabolism. As the gonads mature, the *per* expression was the highest in the eyestalk. The eyestalk is a key organ for light perception and neurohormone secretion in crustaceans [40]. This expression pattern suggests that *Ec-per* may participate in regulating light-signal input to modulate circadian rhythms in peripheral tissues. During the postpartum recovery period, shrimp undergo physiological preparations for subsequent gonadal development and spawning. In response to factors such as organ aging, decreased environmental adaptability, and increased oxygen consumption [16], *Ec-per* expression was significantly upregulated in the gill, intestinal, and gonadal tissues, likely to meet energy requirements. Collectively, these results demonstrate that *Ec-per* exhibits both tissue-specific and developmental stage-dependent expression patterns.

### 4.2. Response of the per to Different Light Colors and Photoperiods

As a core component of the circadian clock genes, *per* plays a crucial regulatory role in the molecular oscillation system of biological rhythms and is involved in modulating the periodic rhythms of various biological activities [41,46]. Our findings indicate that *per* expression responds to white, blue, and red light, peaking at 3 h of light exposure. Following this peak, expression continued to oscillate, suggesting sensitivity to photic stimulation—a pattern similar to that observed for *cry1* under the same light conditions [47]. In the later stages of *E. carinicauda* aquaculture, exposure to specific light colors (blue or red color) for 3 h (based on light color and RNA interference results) may promote gonadal development in shrimp. Previous studies have shown that CRY1 participates in circadian regulation by sensing blue and purple light signals [48], and that CRY1-PER products can form heterodimers that translocate into the nucleus to exert their function [49]. Blue light is thought to activate the CRY protein, which interacts with the PER-TIM heterodimer, leading to its degradation or functional inhibition, thereby resetting the light signal and synchronizing the circadian oscillator. This process ultimately influences the transcriptional rhythm of the *per* gene. Thus, we speculated that *per* may cooperate with *cry1* in a signaling cascade to mediate responses to changes in external light color, though the precise mechanisms warrant further investigation.

To examine the photoperiod response of *per*, its expression profile was analyzed under various light conditions. Results showed that the *per* expression level exhibited regular oscillations under various light conditions, including continuous darkness. The pattern of alternating peaks and troughs was consistent with previous reports [50,51]. Light not only elicits acute behavioral and physiological responses but also drives phase shifts in the circadian clock. Altered photoperiods reduced amplitude and induced phase shift in the *per* expression rhythm, potentially due to light-mediated effects on the binding efficiency of the PER–TIM complex and the phosphorylation of PER protein. These changes could influence its nuclear translocation timing, thereby shifting the rhythmic phase [52,53]. Overall, the findings indicate *per* as an endogenous circadian clock gene whose expression remains largely independent of external light conditions; it may participate in regulating physiological rhythms through interactions with other clock genes.

### 4.3. Function of the per and Its Effect on Ovarian Development in E. carinicauda

After knocking down *per* expression, we found that the *tim* and *cry1* genes were also significantly downregulated, indicating that *per* likely participates in a molecular feedback loop with these genes to synergistically regulate the circadian rhythms. In *Drosophila*, CLK and CYC heterodimers bind to the E-box (CACCTG), activating the transcription of core clock genes, *per* and *tim*, and driving circadian oscillations in the clock genes expression [54]. The resulting PER and TIM proteins form heterodimers that translocate into the nucleus and inhibit CLK/CYC activity, thereby repressing their own transcription in a negative feedback loop [55]. In mammals, the co-regulatory molecular mechanism mediated by the PER-TIM-CRY complex is highly conserved [56,57,58]. CLK/CYC and its mammalian homolog CLK/BMAL1 serve as central transcriptional activators in the circadian molecular network.

In *M. nipponense*, it was first reported that clock genes are involved in the ovarian development of crustaceans, revealing that the circadian function in the brain is closely related to ovarian development and female reproduction [28]. To further explore the role of *per* in ovarian development, its expression profiles were analyzed across five ovarian developmental stages. Our results indicated that *per* expression progressively increased prior to gonadal maturation, declined significantly after maturation, and peaked following ovulation. This expression pattern resembles that of the *EcR* gene in ovarian tissue [59], which has been reported to play a critical regulatory role in ovarian maturation [59]. Interfering with the *per* gene also reduced *EcR* mRNA levels, further supporting the involvement of *per* in regulating gonadal developmental cycles [59]. Beyond interactions between photoperiod-driven circadian rhythms and ovarian estrogen signaling, coordinated multi-system regulation is required for reproduction [60]. Since female shrimp rely on mating to complete spawning [61], we propose that the *per* gene integrates photoperiodic cues with endocrine pathways to regulate ovarian maturation rhythms and synchronize spawning events.

In *D. melanogaster*, core circadian clock genes (such as *per*, *tim*, *Clk*, and *cyc*) are crucial for reproductive fitness, and deficiencies in these genes can result in defective sperm incapable of fertilization [62]. This is likely due to disrupted circadian rhythms, which impair the energy and material supply necessary for yolk synthesis [63]. In our study, interference of the *per* gene downregulated reproductive-related genes, including *EcR*, potentially delaying ovarian development. We speculate that circadian clock genes do not directly regulate ovarian development processes or downstream hormone cascades, but instead act upstream by mediating photoperiodic timing [64]. The loss of *per* may thus disrupt circadian rhythmicity, leading to altered ecdysteroid secretion dynamics. This disruption may be through a transcriptional feedback loop involving coordinated regulation among these genes. Notably, even under short-day conditions in insects, RNAi-mediated *per* knockdown can promote ovarian development. ISH further revealed that the *per* gene could be detected in oocytes, including exogenous and endogenous yolk-synthesizing stages.

This study demonstrates rhythmic synchrony between the circadian gene *per* and key reproductive gene *EcR* in the ridgetail white prawn, offering key evidence for the coordination of biological clock and reproduction in crustaceans. While the observed correlation does not establish causation, *EcR* expression may be regulated by the circadian system or involved in reproductive feedback regulation, warranting further functional validation. The findings support the conservation of the “clock–endocrine–reproduction” axis across arthropods, while phase-specific expression and photoperiod sensitivity likely reflect habitat adaptation in shrimp. Comparative studies with other crustaceans could help distinguish conserved mechanisms from species-specific adaptations. In summary, this work outlines a molecular profile of circadian–reproductive coupling in the ridgetail white prawn, providing a foundation for mechanistic and cross-species studies, with implications for understanding crustacean reproductive adaptation and aquaculture management.

This study has certain limitations. First, the RNAi approach employed does not fully abrogate target gene function, and residual activity may lead to phenotypic effects weaker than those of a complete knockout. Second, while our findings indicate interactions among the *per, tim,* and *cry* genes, the precise molecular mechanisms linking these genes and their downstream pathways remain unclear. For instance, whether *per* directly regulates *EcR* to influence ovarian development or acts indirectly through the PER-TIM-CRY complex requires further investigation. Additionally, future studies should examine whether the disruption of *per* alters circadian rhythms in shrimp and further assess ovarian development status through gonadal indices and related gene expression profiles.

To address these limitations, future research studies should: (1) use gene editing technologies such as CRISPR/Cas9 to generate homozygous mutants [65,66,67] to validate and deepen current findings; (2) apply ChIP-seq to delineate the regulatory network of the PER–TIM–CRY protein complex and identify its potential downstream targets.

## 5. Conclusions

In this study, we first cloned the full-length cDNA sequences of the *per* gene from *E. carinicauda*. Our findings demonstrate that *per* plays a critical role in embryonic and larval development, with its tissue expression varying across developmental stages. Under different photoperiods, *per* exhibited phase shifts and altered oscillation amplitude, suggesting that the shrimp adjust their physiological activities in response to light cycles. Knockdown of the *per* gene resulted in significant downregulation of *tim* and *cry1*, as well as suppression of the *EcR* gene. ISH results further indicated that *per* is involved in oocyte proliferation and the accumulation of exogenous nutrients. Together, these results offer new insights into modulating circadian rhythms and reproduction in crustaceans through optimized light management.

## Figures and Tables

**Table 1:** Primer sequences used in this study.

Primer Name	(5′-3′) Primer Sequence	Purpose
*per*-1600-F	ACCCGAGGAAGTTATGAAGG	Core sequence amplification
*per*-2240-R	GCGAATTTGTCTGCGACTG	
*per*-2231-F	ATTGGACGTTCGTATCGG	
*per*-3289-R	CTCCTGTTTCCCTGGTGA	
*per*-3063-F	GGTCTGCTCGTGGTAGTAATG	
*per*-3641-R	GTTCATCCACCAAAGAGGG	
*per*-3267-3	AGAGCATTGGATCACCAGGGAAACAG	3′RACE amplification
*per*-3473-3	CCACGCTCAAAATGCCCTCGTCCTA	
*per*-2061-5	TCCATCTTACTGTCTCCACTTTCATCGG	5′RACE amplification
*per*-2197-5	CCCATTTCCAGTGCTGTCTGATTGTCT	
*per*-1152-5	CACCAGAACTTGAGCCTTCGCCATC	
*per*-1615-5	ATAACTTCCTCGGGTAGCGGCGTCA	
*per*-siRNA-A	GATCACTAATACGACTCACTATAGGGGCTGCCTAATGTGGTTTATTT	siRNA interference
*per*-siRNA-B	AAATAAACCACATTAGGCAGCCCCTATAGTGAGTCGTATTAGTGATC	
*per*-siRNA-C	AAGCTGCCTAATGTGGTTTATCCCTATAGTGAGTCGTATTAGTGATC	
*per*-siRNA-D	GATCACTAATACGACTCACTATAGGGATAAACCACATTAGGCAGCTT	
*Ec*-*per*-F	CCTGAACCGCCACCTAATGT	qRT-PCR
*Ec*-*per*-R	GTCTGTGGGCAACTTCCGTA	
*Ec*-*tim*-F	CACAGGTTCCCTCAGAACCC	
*Ec*-*tim*-R	GGGATGATGTACTGGAGCGG	
*Ec*-*cry*1-F	AGAAGAGTGAACAGAGGGAAGC	
*Ec*-*cry*1-R	GGTAGCCCCAGTAAAGACGA	
*Ec*-*EcR*-F	GGCTACCATTACAACGCCCT	
*Ec*-*EcR*-R	CTCGGCGATCCTTTAGGCTT	
*Ec*-18S-F	TATACGCTAGTGGAGCTGGAA	Internal reference
*Ec*-18S-R	GGGGAGGTAGTGACGAAAAAT	
*per*-RO-F	CAGGTCGACTCTAGAGGATCCATGAATATGGAAGGAAGTGATGGCC	Homologous recombination primers for overexpression
*per*-RO-R	ACTATAGGGAGACCGGAATTCTCAAGATGAATAACCTGAAGATTCTGC	
*Ectim*-O-F	TAATACGACTCACTATAAGGCAGGTCGACTCTATGAATATGGAAGGAAGTGATGGC	Overexpression
*Ectim*-O-R	ACTATAGGGAGACCGGAATTCTCAAGATGAATAACCTGAAGATTCTGC	
*per*-RH-F	CAGGTCGACTCTAGAGGATCCGAATACAAGCTTGCATGCCTGC	Homologous recombination primers for in situ hybridization
*per*-RH-R	ACTATAGGGAGACCGGAATTCTATAGTGTCACCTAAATCGTATGTGTATGA	
*Ectim*-SP6-F	ATTTAGGTGACACTATAGAATACAAGCTTGCATGCCTGCAGGTCGACTCTAGAGTCTGA	In situ hybridization
*Ectim*-T7-R	TAATACGACTCACTATAGGGAGACCGGAATTCCAGACCCAGAGCAAGACTG	

## Data Availability

All data in this study are included in the article.
